# Genistein Suppresses LPS-Induced Inflammatory Response through Inhibiting NF-κB following AMP Kinase Activation in RAW 264.7 Macrophages

**DOI:** 10.1371/journal.pone.0053101

**Published:** 2012-12-31

**Authors:** Guiyuan Ji, Yupei Zhang, Qinhe Yang, Shaobin Cheng, Jing Hao, Xihong Zhao, Zhuoqin Jiang

**Affiliations:** 1 Guangdong Provincial Institute of Public Health, Guangzhou, China; 2 Guangdong Provincial Center for Disease Control and Prevention, Guangzhou, China; 3 Department of Traditional Chinese Medicine, Medical College of Jinan University, Guangzhou, China; 4 Guangxi Botanical Garden of Medicinal Plant, Nanning, China; 5 Key Laboratory for Green Chemical Process of Ministry of Education, School of Chemical Engineering and Pharmacy, Wuhan Institute of Technology, Wuhan, China; 6 Department of Nutrition, School of Public Health, Sun Yat-Sen University, Guangzhou, China; Temple University School of Medicine, United States of America

## Abstract

Genistein, the major isoflavone in soybean, was recently reported to exert beneficial effects in metabolic disorders and inflammatory diseases. In the present study, we investigated the effects and mechanisms of a dietary concentration of genistein on the inflammatory response in lipopolysaccharide (LPS)-treated RAW 264.7 macrophages. Our results demonstrated that genistein effectively inhibited the LPS-induced overproduction of tumor necrosis factor-alpha (TNF-α) and interleukin 6 (IL-6), as well as LPS-induced nuclear factor kappa B (NF-κB) activation. In addition, the data also showed that genistein prevented LPS-induced decrease in adenosine monophosphate-activated protein kinase (AMPK) phosphorylation. These effects were obviously attenuated by an AMPK inhibitor. Taken together, our results suggest that the dietary concentration of genistein is able to attenuate inflammatory responses via inhibition of NF-κB activation following AMPK stimulation. The data provide direct evidence for the potential application of low concentrations of genistein in the prevention and treatment of inflammatory diseases.

## Introduction

Accumulating evidence suggests that chronic inflammatory processes are involved in the pathogenesis of common metabolic disorders, such as lipid dysregulation, obesity, atherosclerosis, and insulin resistance [1], [2], [3], [4]. Macrophages play a central role in organizing the release of inflammatory mediators, including nitric oxide (NO), tumor necrosis factor-alpha (TNF-α), and interleukin-6 (IL-6) [5], [6]. Due to their highly reproducible response to lipopolysaccharide (LPS), the RAW 264.7 mouse macrophage cell line is widely used for inflammation studies.

Adenosine monophosphate-activated protein kinase (AMPK) has been postulated to respond to intracellular AMP levels or the AMP: ATP ratio [7]. It is an evolutionary conserved serine/threonine kinase that regulates cellular energy homeostasis [8], [9]. AMPK activation reportedly requires phosphorylation of Thr-172 on the α-subunit by upstream kinases, such as liver kinase B1(LKB1) or Ca2^+^/calmodulin-dependent protein kinase kinase-II (CaMKK II) [10]. Recent studies have demonstrated that AMPK activation can improve metabolic disorders and inflammatory responses, making it an attractive and novel target for treating metabolic syndrome-related diseases [11], [12].

Genistein (4′, 5, 7-trihydroxyisoflavone) is a naturally occurring flavone and the major isoflavone in soybean. It is reported that gensitein has numerous anti-oxidative and anti-cancer effects and is known to inhibit tyrosine-specific protein kinases. Recent studies have demonstrated that the beneficial effects of genistein on metabolic disorders are associated with AMPK activation in liver, muscle, and peripheral tissues [13], [14], [15], [16]. We previously showed that genistein administration has a significant anti-inflammatory effect on high-fat diet-induced nonalcoholic steatohepatitis (NASH) rats [17]. However, the molecular mechanisms underlying genistein-mediated inflammatory response suppression are not fully understood, and dosages (20–200 µM) [18], [19], [20] used in other in vitro studies are usually much higher than the concentration levels (0.01–10 µM) [21], [22], [23], [24] that are found in human plasma after digestion.

In the present study, we investigated whether dietary concentrations of genistein (1, 5, 10 µM) could attenuate inflammatory responses in LPS-treated RAW264.7 macrophages, and if so, how it exerted these effects.

## Materials and Methods

### Materials

Genistein was purchased from Cayman Chemical Company (Michigan, USA). ELISA kits for IL-6 and TNF-α quantification were purchased from R&D Systems (Boston, USA). LPS, 5-Aminoimidazole-4-carboxamide-1-β-D-ribofuranoside (AICAR) and AMPK inhibitor Compound C (Com C) were obtained from Sigma (St Louis, MO, USA). The antibodies for GAPDH, NF-κB p65 and Histone 2H.X were obtained from Santa Cruz Biotechnology (Santa Cruz, CA, USA), and antibodies against IκB-α, AMPK, phospho-IKKα/β, phospho-AMPK (Thr172) were purchased from Cell Signaling Technology (Beverly, MA, USA). Reverse transcriptase kit was purchased from Fermentas Inc (Glen burnie, MD, USA) and SYBR Green Master Mix was obtained from GeneCopoeia Inc (Maryland, USA). Enhanced Chemiluminescent (ECL) was obtained from Pierce Biotechnology (Rockford, IL, USA). TRIzol reagent was obtained from Invitrogen (Carlsbad, CA, USA).

### Cell Culture and Treatment

RAW 264.7 mouse macrophage cells were obtained from the Cell Bank of Type Culture Collection of Chinese Academy of Sciences, Shanghai Institute of Cell Biology. Cells were cultured as described by Nan Huang et al. [25] in Dulbeco's Modified Eagle Medium (DMEM) supplemented with 10% fetal bovine serum, 1.5 g/L sodium bicarbonate and 100 IU/mL penicillin/streptomycin at 37°C in a 95% humidified atmosphere with 5% CO_2_. Cells were plated at 1.5×10^5^ cells/mL in a plate with 48/96 wells. Genistein were dissolved in dimethyl sulfoxide (DMSO) and diluted 1∶1000 in culture medium. Controls were treated with the vehicle (0.1% DMSO). The tested genistein were dissolved in DMSO at a concentration of 10 mM and stored frozen in small aliquots until used. The compounds were diluted with supplemented DMEM as needed, before cell exposure. Cells were incubated with various concentrations of genistein (1, 5 or 10 µM) or positive/negative chemicals (AICAR, Com C) and then stimulated with LPS (1 µg/ml) for the indicated time. AICAR or Com C was also dissolved in DMSO.

### MTT Assay

The mitochondrial-dependent reduction of 3-(4,5-dimethylthizaol-2yl)-2,5-diphenyl tetrazolium bromide(MTT) to formazan was used to measure cell respiration as an indicator of cell viability [26]. Briefly, RAW 264.7 cells were seeded onto 96-well plates (10^5^ cells/well) and grow to confluence in DMEM. Genistein was dissolved in DMSO, and the DMSO concentrations in all assays did not exceed 0.1%. After 24 h incubation, cells were pretreated in triplicate with or without indicated concentrations (0 µM, 1 µM, 5 µM, 10 µM, 50 µM and 100 µM) of genistein for 1 h, then were incubated with 1 µg/mL LPS for 12 h, 24 h and 48 h respectively. After LPS incubation, the medium was removed, the cells were washed, and 0.5 mg/mL of MTT was added to each well and incubated for another 4 h at 37°C. After removing the supernatant, 150 µL DMSO was added to the cells to dissolve the formazan. The absorbance of each group was measured by using a microplatereader at wavelength of 570 nm. The control group consisted of untreated cells was considered as 100% of viable cells.

### Enzyme Linked Immunosorbent Assay (ELISA)

RAW 264.7 macrophages were cultured in 96-well plate with or without different genistein concentrations (0.1 µM, 1 µM, 5 µM and 10 µM) for 1 h, and then incubated with or without 1 µg/mL LPS for 24 h. Supernatants were obtained and frozen at 80°C until analysis. IL-6 and TNF-α in the culture medium were determined by ELISA kit according to the manufacturer’s recommendations. Both TNF-α and IL-6 were measured in triplicate, and the ELISA plates were read using a microplate reader (LX Bio-Tec Instruments, USA).

### Quantitative Real-time Reverse Transcriptase Polymerase Chain Reaction (qRT-PCR)

RAW 264.7 macrophages were pretreated with different concentration of genistein(0.1 µM to 10 µM) for 1 h, and then incubated with 1 µg/ml LPS for 24 h.

Cell homogenization, RNA extraction, reverse transcription, and quantitative PCR were performed as described [27]. Total RNA was isolated from RAW 264.7 macrophages by using Trizol reagent, and then transcribed into cDNA with reverse transcriptase kit. Real-time polymerase chain reaction was performed with the SYBR green method and evaluated in an iCycler detection system (Bio-Rad, Hercules, CA). Primers specific for mouse TNF-α, IL-6 and β-actin, which sequences were shown in Table 1, were designed using the Primer Express™ design software (Applied Biosystems). For PCR, the amplification was performed for 40 repetitive thermal cycles with SYBR green (95°C for 10 s, 60°C for 20 s, and 72°C for 15 s, followed extension at 72°C for 10 min). The relative expression ratio (R) of a target gene was expressed for the sample versus the control in comparison to the β-actin. The values of threshold cycle (Ct) were determined by automated threshold analysis using Opticon Monitor 3.1 software. The relative levels of each gene expression were determined by the 2^−ΔΔCt^ method. ΔΔCt was (Ct_target_ – Ct_β-actin_) _treatment_ – (Ct_target_ – Ct_β-actin_) _control_.

**Table 1:** Primer sequences used to amplify cDNA for qRT-PCR.

mRNA	Forward Primer	Reverse Primer	Accession No.
Mouse IL-6	acaaccacggccttccctactt	gtgtaattaagcctccgact	NM-031168
Mouse TNF-α	tggagtcattgctctgtgaaggga	agtccttgatggtggtgcatgaga	NM-013693
Mouse β-actin	tactgccctggctcctagca	tggacagtgaggccaggatag	NM-031144

### Western Blot Analysis

An equal amount of protein (50 µg) from each sample was separated on 10% SDS-polyacrylamide gel electrophoresis (SDS-PAGE), and then was transferred onto equilibrated polyvinylidene difluoride membranes (Millipore USA). The bolts were blocked with 5% nonfat milk in tris-buffered saline with 0.1% Tween-20 (TBST, 25 mM Tris, 137 mM NaCl, 2.7 mM KCl, and 0.1% Tween-20) at room temperature for 2 h, and then incubated overnight at 4°C with specific primary antibody. After being washed with TBST three times, the blots were hybridized with secondary antibodies conjugated with horseradish peroxidase for 1 h at room temperature. The antibody-specific protein was visualized by ECL detection system.

### Statistics

The values are expressed as the mean ± standard error of the mean (S.E.M). Comparisons between the different treatment groups were analyzed via one-way ANOVA and the least significant difference (LSD), and differences were considered significant at *P*<0.05. All calculations were performed with SPSS 13.0 statistical software (Chicago, USA).

## Results

### RAW 264.7 Macrophage Cell Viability

**Figure 1:**
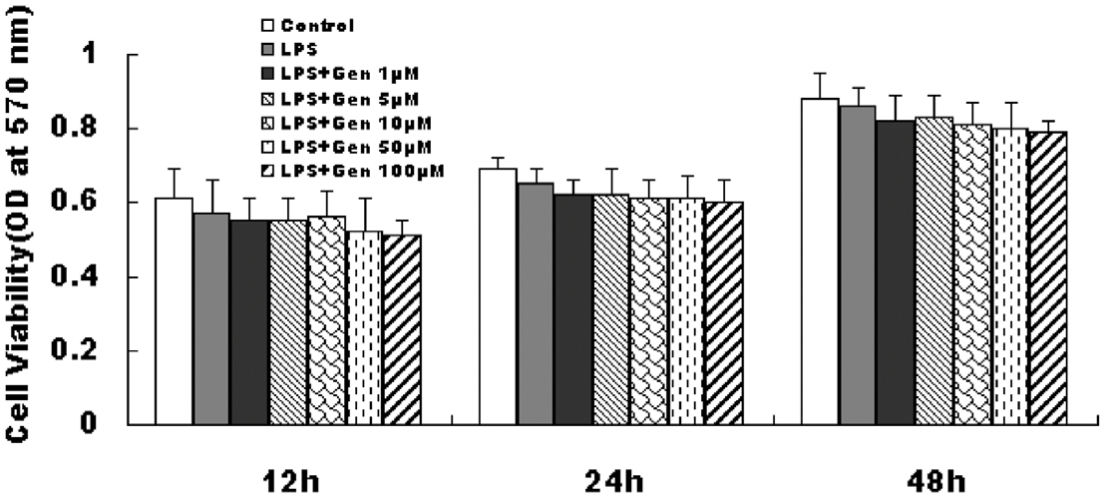
Effect of genistein and LPS on RAW 264.7 macrophage viability. Cells were pretreated with genistein (0 µM to 100 µM) for 1 h, then incubated with or without 1 µg/mL LPS for 12 h, 24 h, or 48 h respectively. Cell viability was determined by MTT assay. Data are the mean ± S.E.M (n = 3 ) of three independent experiments.

RAW 264.7 cells were treated with 1, 5, 10, 50, or 100 µM genistein for 1 h and then incubated with 1 µg/mL LPS for 12, 24, or 48 h. MTT assay did not show any significant difference in RAW 264.7 macrophage viability among the control and genistein-treated groups, suggesting that genistein is not cytotoxic (*P*>0.05) (Fig. 1).

### Effect of Genistein on IL-6 and TNF-α Levels in LPS-stimulated RAW 264.7 Cells

**Figure 2:**
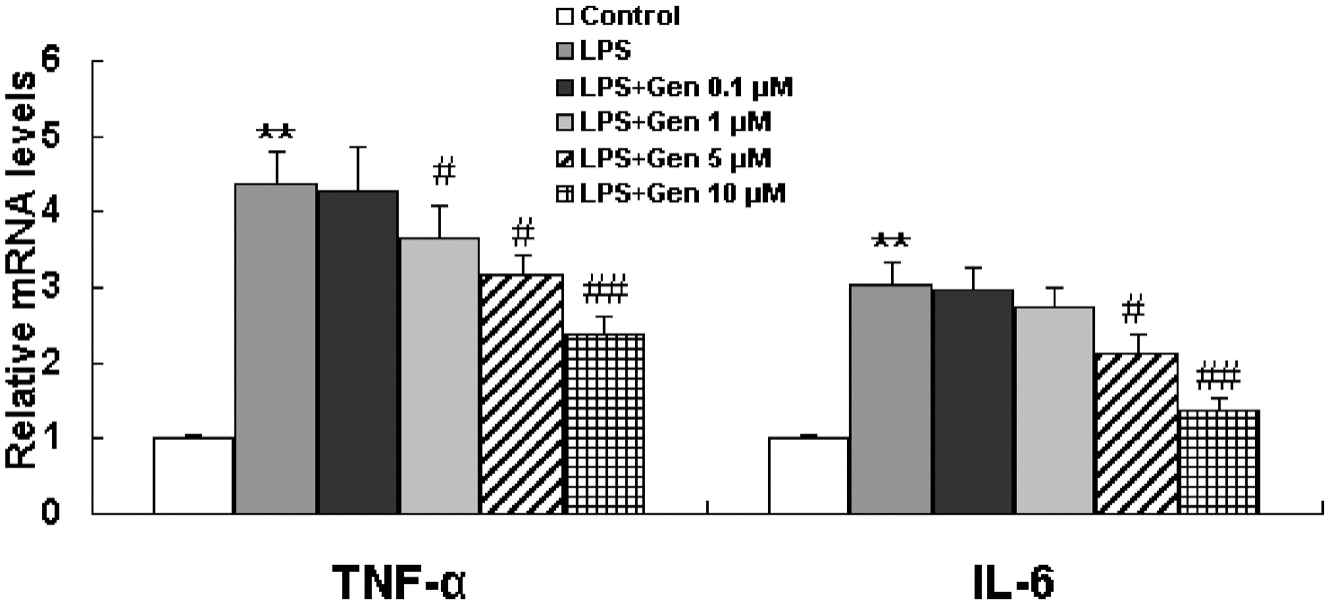
Inhibitory effect of genistein on TNF-α and IL-6 mRNA levels in LPS-treated RAW 264.7 macrophages. Cells were pretreated with genistein (0.1 to 10 µM) for 1 h and then incubated with LPS (1 µg/mL) for 24 h. Cells were collected, and IL-6 and TNF-α mRNA levels were determined by qRT-PCR and normalized to β-actin. Each column represents the mean ± S.E.M of triplicate experiments. **P<0.01 vs. control, ^##^ *P*<0.01,^ #^ *P*<0.05 vs. LPS alone.

Elevated levels of inflammatory cytokines are considered to be biomarkers of inflammation. RAW 264.7 macrophages were incubated with genistein (0.1, 1, 5, or 10 µM) in the presence or absence of LPS (1 µg/mL). ELISA assay and qRT-PCR were applied to examine the effect of genistein on IL-6 and TNF-α levels in LPS-treated cells. As shown in Fig. 2 and Table 2, LPS alone dramatically increased mRNA and protein levels of IL-6 and TNF-α. The results showed that 0.1 µM genistein had no obvious effect on LPS-induced TNF-α mRNA and protein overproduction, however, higher doses of genistein(1,5 and 10 µM) reduced IL-6 and TNF-α mRNA and protein levels in a dose-dependent manner. In addtion, the results showed that genistein (0.1 µM to 10 µM) alone showed no effect on cytokine levels in LPS-untreated cells.

**Table 2:** Effect of genistein on TNF-α and IL-6 production in LPS-treated RAW 264.7 cells.

	TNF-α (pg/ml)	IL-6 (pg/ml)
Control	132.6±29.74	103.27±26.83
LPS	4069.38±455.81**	401.28±42.19**
LPS+Gen 0.1 µM	4048.84±431.27	405.76±43.22
LPS+Gen 1 µM	3626.64±405.29#	379.57±38.92
LPS+Gen 5 µM	3226.82±338.24^##^	321.72±34.55#
LPS+Gen 10 µM	2438.57±259.83^##^	257.96±24.81^##^
Gen 0.1 µM	140.43±32.85	98.72±19.23
Gen 1 µM	134.58±20.71	100.45±21.37
Gen 5 µM	135.18±18.43	104.86±20.24
Gen 10 µM	129.26±18.62	102.97±21.55

Values are the mean ± S.E.M (n = 3) of three independent experiments.

* *P*<0.05, **P<0.01 compared to the control group.

# *P*<0.05, ^##^ *P*<0.01 compared to the LPS group.

### Effects of Genistein on NF-κB Activation in LPS-stimulated RAW 264.7 Cells

**Figure 3:**
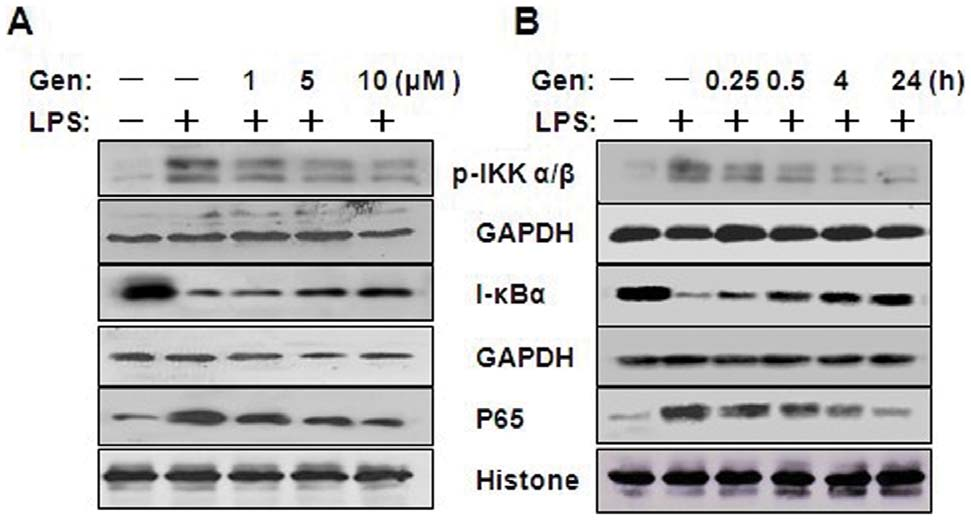
Effect of genistein on AMPK activation in LPS-treated RAW 264.7 macrophages. (A) Cells were pretreated with 1, 5, or 10 µM genistein for 1 h and then incubated with 1 µg/mL LPS for 24 h. (B) Cells were pretreated with 10 µM genistein for 1 h and then incubated with 1 µg/mL LPS for 0.25, 0.5, 4, or 24 h. Cell lysates were prepared and analyzed for AMPK and p-AMPK by western blotting. GAPDH was used as an internal control. Experiments were repeated three times, and representative blots are shown here.

To further characterize the mechanism underlying the anti-inflammatory effects of genistein, we assessed the NF-κB pathway, which is critical in the inflammatory response. In an inactivated state, NF-κB p65 is localized in the cytosol where it is complexed with its inhibitor, IκB, which can be phosphorylated by proinflammatory cytokines, LPS, or growth factors, and then ubiquitinated and rapidly degraded. Activated NF-κB p65 is released and translocates into the nucleus where it binds to specific DNA sequences to induce target genes expression. We therefore examined the effect of genistein on nuclear p65 protein levels in RAW 246.7 cells treated with LPS. As shown in Fig. 3, LPS induced large increase in the content of nuclear p65 protein, however, genistein prevents the increase in a dose- (Fig. 3A) and time-dependent (Fig. 3B) manner. Next, we investigated whether genistein inhibits LPS-induced degradation of IκB-α in RAW 264.7 macrophages by Western blotting with anti-IκB-α antibody. Fig. 3 shows that LPS-induced IκB-α degradation was obviously blocked by pretreatment with genistein. Because IKK-α and β are the upstream kinases of IκB in the NF-κB signal pathway [28], we also measured the effect of genistein on LPS-induced IKK-α/β activation by western blotting. LPS was found strongly induce IKK-α/β phosphorylation, whereas genistein inhibited this phosphorylation in a concentration- (Fig. 3A) and time-dependent (Fig. 3B) manner. The data indicates that genistein could inhibit LPS-stimulated NF-κB activation in RAW 264.7 macrophages.

### Effects of Genistein on AMPK Activation in LPS-stimulated RAW 264.7 Cells

Recent studies have revealed that genistein is capable of activating AMPK in adipocytes and hepatocytes, and AMPK pathway correlates with inflammatory disease [14], [29]. However, it is unclear whether genistein is also able to activate AMPK in stimulus induced inflammatory response in macrophages.

**Figure 4:**
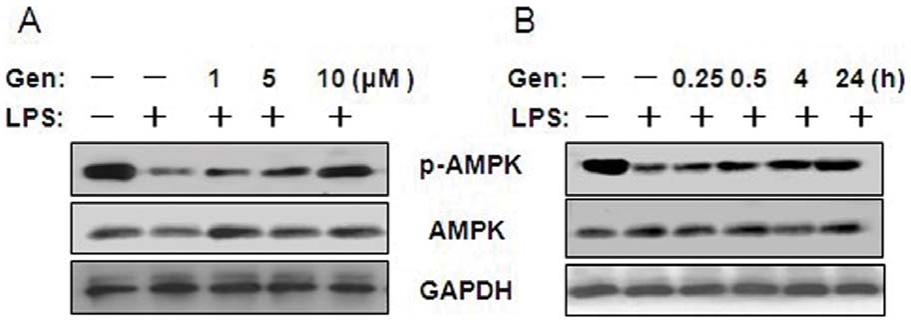
Effect of genistein on NF-κB activation in LPS-treated RAW 264.7 macrophages. (A) Cells were pretreated with 1, 5,or 10 µM genistein for 1 h, and then incubated with 1 µg/mL LPS for 24 h. Cells were pretreated with 10 µM genistein for 1 h, and then incubated with 1 µg/mL LPS for 0.25, 0.5, 4, or 24 h. (B) Cell lysates were prepared and analyzed for IκB-α, p-IKKα/β, or GAPDH by western blotting. The nuclear fraction was collected for assessment of NF-κB p65 and histones. GAPDH or histone was used as an internal control. Experiments were repeated three times, and representative blots are shown here.

We studied the dose-dependent effect of genistein on AMPK activation in LPS-treated cells and found that LPS alone decreased AMPK phosphorylation, whereas pretreatment with genistein (1 to 10 µM) prevented this effect (Fig. 4A). The time-dependent effect study demonstrated that pretreatment with genistein (10 µM) increased AMPK phosphorylation in LPS-treated cells after 0.25 h incubation, and phosphorylation levels increased correspondingly as incubation time increased (Fig. 4B).

### Genistein Inhibits IL-6 and TNF-α Production via AMPK Activation

To further explore whether AMPK activation is involved in IL-6 and TNF-α production, we assessed the effects of AMPK agonist AICAR and AMPK inhibitor Com C. As shown in Table 3, pretreatment with either AICAR (1 mM), or 10 µM genistein obviously inhibited LPS-induced TNF-α and IL-6 overproduction. However, Com C pretreatment significantly attenuated the inhibitory effects of genistein on cytokine generation in LPS-treated cells. AICAR or Com C alone showed no obvious effect on cytokine production in control cells. The data indicate that the inhibitory effect of genistein on LPS-induced TNF-α and IL-6 overproduction is dependent on AMPK activation.

**Table 3:** Effect of genistein, AMPK activator, and AMPK inhibitor on TNF-α and IL-6 production.

	TNF-α (pg/ml)	IL-6 (pg/ml)
Control	128.03±14.85	110.56±18.91
LPS	4103.16±418.27**	392.74±45.83**
AICAR	129.56±15.46	106.63±20.82
Gen 10 µM	132.01±16.58	110.36±18.55
LPS +AICAR	2236.09±237.14^##^	245.76±25.42^##^
Com C	129.26±18.62	102.97±21.55
LPS+ Com C	4452.24±427.96	421.29±43.65
LPS+Gen 10 µM	2647.28±272.42^##^	273.84±25.55^##^
LPS +Gen 10 µM+Com C	3308.47±304.75^#△^	314.72±30.81^#△^

LPS+AICAR: cells were pretreated with 1 mM AICAR for 1 h and then incubated with 1 µg/mL LPS for 24 h; LPS+Gen 10 µM: cells were pretreated with 10 µM genistein for 1 h and then incubated with LPS for 24 h; LPS+Com C: cells were pretreated with 20 µM Compound C for 30 min and then incubated with LPS for 24 h; LPS+Gen 10 µM+Com C: cells were pretreated with 20 µM Compound C for 30 min, incubated with 10 µM genistein for 1 h, and then co-cultured with LPS for 24 h. Values are mean ± S.E.M of three independent experiments.

* *P*<0.05,^ **^ *P*<0.05 compared to the control group.

# *P*<0.05, ^##^ *P*<0.01 compared to the LPS group.

△ *P*<0.05 compared to the LPS+Gen 10 µM group.

△ *P*<0.05 compared to the LPS+Gen 10 µM group.

### AMPK Stimulation Suppresses NF-κB Activation

As mentioned above, both AMPK and NF-κB pathways were involved in genistein’s anti-inflammatory effects. To further evaluate the relationship between AMPK and NF-κB activation in LPS-induced inflammatory response in macrophages, AMPK agonist AICAR and inhibitor Compound C were aplied in the following experiment.

**Figure 5:**
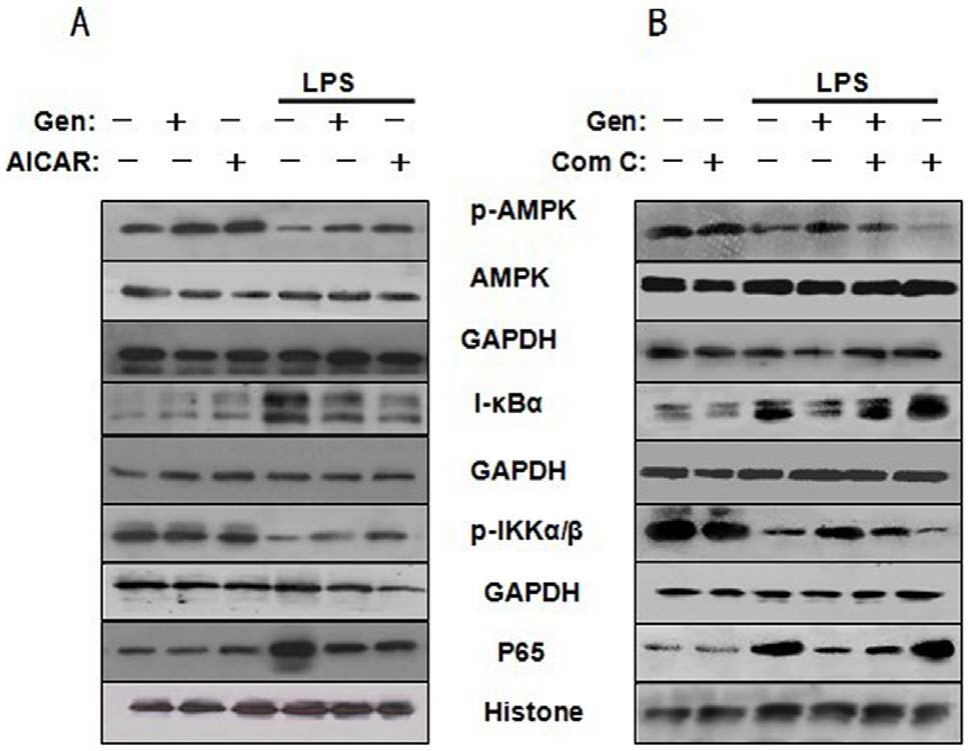
AMPK activation decreases the inhibitory effect of genistein on NF-κB activation in LPS-treated RAW 264.7 macrophages. (A) Cells were pretreated with or without 10 µM genistein or 1 mM AICAR for 1 h and then incubated with 1 µg/mL LPS for 24 h. (B) Cells were pretreated with or without 20 µM Compound C for 30 min, then with or without 10 µM genistein for 1 h and stimulated with LPS for 24 h. Cytoplasm and nuclear extracts were collected for determination of AMPK, p-AMPK, I-κBα, p-IKKα/β, GAPDH, p65, and histone by western blotting. GAPDH or histones was used as an internal control. Experiments were repeated three times, and representative blots are shown here.

As shown in Fig. 5A, western blot analysis showed that 10 µM genistein or 1 mM AICAR alone increased AMPK phosphorylation in LPS-untreated cells, however, neither treatment affected total AMPK expression, I-κBα degradation, nulear p65 content, or p-IKKα/β phosphorylation. Pretreatment with both 1 mM AICAR and 10 µM genistein increased AMPK phosphorylation but decreased I-κBα degradation, IKKα/β phosphorylation and nuclear p65 content in LPS-treated cells. As shown in Fig. 5B, Com C alone had no obvious effect on the above parameters in LPS-untreated cells. However, pretreatment with Com C aggravated LPS-induced AMPK inactiavation, IKKα/β phosphorylation,I-κBα degradation and increase in nuclear p65 protein level. The result also showed that Com C pretreatment blocked the inhibitory effect of genistein on LPS-induced I-κBα degradation, IKKα/β phosphorylation and increase in p65 level. Collectively, the data indicate that genistein suppresses LPS-induced NF-κB activation following AMPK activation.

## Discussion

Recent reports suggest that chronic low-grade inflammation may be an effective target for treating metabolic syndrome-associated diseases. This study demonstrates for the first time that a dietary concentration of genistein can attenuate proinflammatory responses in LPS-treated macrophages by inhibiting NF-κB activation following AMPK stimulation. These results may have implications in the prevention and treatment of chronic low-grade inflammatory condition accompanied diseases.

We showed that dietary concentrations of genistein (1, 5, and 10 µM) inhibited LPS-induced TNF-α and IL-6 overproduction, confirming that genistein is an anti-inflammatory substance. We previously demonstrated that genistein administration prevented liver dysfunction, attenuated NASH progression, and decreased levels of inflammatory factors (TNF-α and IL-6) in plasma and liver of NASH model rats [17]. Our present findings also confirmed previous reports that genistein could suppress the inflammatory response in various cell lines and animal studies. Chunyeon et al. [18] demonstrated that 50 and 100 µM genistein significantly reduced LPS-induced NO production, reduced thiobarbituric acid-reactive substances (TBARS) accumulation, increased antioxidant enzyme activity, and suppressed NF-κB activation in Raw 264.7 macrophages. It was found that 50 µM isoflavones (genistein, daidzein, and glycitein) could inhibit LPS-induced NO production and decrease iNOS activity and gene expression in RAW 264.7 macrophages [20]. The present results also indicate that genistein can inhibit LPS-induced inflammatory cytokine overproduction in macrophages. An obvious difference between our study and previous in vitro studies was that we used lower genistein dosages (1, 5, and 10 µM) than other studies (20 to 200 µM). It is reported [21], [22], [30] that total genistein content in human plasma is usually less than 10 µM. However, genistein concentration in liver can be more than 10 µM because there is a first-pass effect in the liver. The present study showed that even dietary concentrations of genistein (1, 5, and 10 µM ) can exert an anti-inflammatory effect.

Genistein is the major active isoflavonoid in soybean. Its anti-cancer and anti-inflammatory effects have been documented, but the mechanisms underlying these effects are not fully understood. NF-κB is a pleiotropic regulator of many proinflammatory cytokines and has been found to be activated by a variety of stimuli. The present study demonstrated that genistein (1, 5, and 10 µM) could suppress NF-κB activation in LPS-treated macrophages, which is in agreement with observations that genistein inhibits NF-κB activation in several cell types and stressed animals [31], [32], [33]. The results suggest that genistein’s ability to inhibit LPS-induced TNF-α and IL-6 release may be explained in part by blocking NF-κB activation.

Genistein can reportedly activate AMPK in vivo and in vitro. Hwang et al. [14] demonstrated that 100 µM genistein could inhibit adipocyte differentiation via AMPK activation. Cederroth and colleagues [13] found that dietary phytoestrogens activate AMPK in adipocyte and muscle with subsequent improvement in lipid and glucose metabolism. The present study confirmed that genistein can activate AMPK in LPS-treated macrophages. The present study found that AICAR could also inhibit LPS-induced TNF-α and IL-6 overproduction and NF-κB activation, whereas Com C seemed to play an opposite role. We found that AMPK phosphorylation in LPS-treated RAW 264.7 cells was clearly lower than that in LPS-untreated cells, which is consistent with a previous study. Sag and colleague [34] demonstrated that stimulating macrophages with anti-inflammatory cytokines (e.g., IL-10 and TGF-β) resulted in rapid AMPK phosphorylation/activation, whereas stimulating macrophages with a proinflammatory stimulus (LPS) resulted in AMPK dephosphorylation/inactivation. Although many studies have demonstrated that genistein can activate AMPK, few have explored the mechanism in detail. Hsu and colleague [35] showed that both genistein and resveratrol could activate AMPK, however, the two compounds exerted this effect through different mechanisms. Genistein phosphorylated AMPK Thr-172 via upstream kinase Ca^2+^/CaMKK, whereas resveratrol acts through upstream kinase LKB1. It is also reported that reactive oxygen species (ROS) generated by genistein is one of the elements responsible for AMPK activation [14].

There are results indicating that AMPK signaling can inhibit NF-κB-induced inflammatory responses system in different cell types. Hattori and colleagues [36], [37] demonstrated that metformin or Cilostazol inhibits cytokine-induced NF-κB activation via AMPK activation in vascular endothelial cells. Green C.J., et al. [38], [39] found that NF-κB signaling can be attenuated by AMPK in human myocytes or rat skeletal muscle cells. AMPK inhibition reportedly accelerates or promotes TNF-α production in free fatty acid- and LPS-treated macrophages [29]. The present study confirmed that NF-κB activation was suppressed following AMPK stimulation in LPS-treated macrophages. It is reported that NF-κB subunits are not direct phosphorylation targets of AMPK, but inhibition of NF-κB signaling is mediated by several downstream targets of AMPK, such as sirtuin 1 (SIRT1), peroxisome proliferator-activated receptor gamma coactivator 1-alpha (PGC-1α), and p53 [40].

In a word, the present study provides new insight into the anti-inflammatory mechanisms of genistein, demonstrating that dietary concentrations of genistein mitigate LPS-induced TNF-α and IL-6 release from activated macrophages through activation of NF-κB via AMPK-dependent mechanisms. In conclusion, these data provide evidence that genistein might be a promising innovative agent for treating low-grade inflammatory conditions that accompany many diseases.

However, AMPK activation (by genistein) cannot completely inhibit NFkB activation and cytokine production, thus indicating that other signal pathways besides AMPK may participate in the anti-inflammatory effect of genistein in LPS-treated macrophages. The particular anti-inflammatory effects of genistein should be explored in future studies.
